# ATP during Early Bladder Stretch Is Important for Urgency in Detrusor Overactivity Patients

**DOI:** 10.1155/2014/204604

**Published:** 2014-05-27

**Authors:** Y. Cheng, K. J. Mansfield, W. Allen, R. Chess-Williams, E. Burcher, K. H. Moore

**Affiliations:** ^1^Department of Urogynaecology, St George Hospital, University of New South Wales, Level 1, W.R. Pitney Building, Sydney, NSW 2217, Australia; ^2^Graduate School of Medicine, University of Wollongong, Wollongong, NSW 2522, Australia; ^3^Faculty of Health Sciences & Medicine, Bond University, Gold Coast, QLD 4229, Australia; ^4^Department of Pharmacology, School of Medical Sciences, University of New South Wales, Sydney, NSW 2052, Australia

## Abstract

ATP is an important mediator of urgency in women with detrusor overactivity (DO). In order to understand how different degrees of bladder stretch elicited ATP release in DO patients compared with controls, sequential aliquots were collected during cystometry and ATP release was measured at each degree of bladder filling, in female patients with DO and controls. In both DO and control groups, ATP release was induced during bladder filling, suggesting that stretch stimulated further ATP release. However, the luminal ATP concentrations were already high at early filling stage (200 mL), which was even greater than those at the later filling stages (400 mL and maximum cystometric capacity, MCC), indicating that a substantial ATP release has been induced during early filling (200 mL) in both DO and controls. In DO, ATP release at 200 mL was significantly higher in those with low first desire to void (FDV) (≤200 mL) than in those with higher FDV (>200 mL); this may suggest that ATP release at early stretch may play an important role in urgency (early sensation) in DO. ATP concentrations remained unchanged after voiding, suggesting that voiding did not further induce ATP release into intraluminal fluid.

## 1. Introduction

The urothelium, which was once regarded as an inert, protective barrier, plays a key role in bladder sensory and motor functions [1]. The hypothesis that the purine nucleotide ATP is a neurotransmitter responsible for nonadrenergic, noncholinergic neurotransmission was proposed in 1972 and termed purinergic signalling [2]. More recently, both animal and human studies show that nonneuronal ATP is released from the bladder urothelium in response to stretch [3–6]. Besides urothelial cells, cultured pig myofibroblasts were also reported to release ATP in response to stretch [3]. In rat bladder, intravesical application of ATP has been shown to induce detrusor overactivity [7].

Urothelial derived mediators, such as acetylcholine and ATP, signal the sensation of bladder fullness to the central nervous system, thus triggering the micturition reflex [8]. Such mediators act upon specific receptors such as P2X receptors located on suburothelial afferent nerves [9], with possible involvement of myofibroblasts [10]. In addition to stretch, ATP is also released from animal bladder urothelial cells and tissue strips in response to capsaicin and acid [3, 5]. In human subjects, OAB was shown to be associated with increased ATP release [11, 12].

Previous studies have explored the hypothesis that ATP in bladder luminal fluid could be a marker for the sensation of “urgency” as experienced by patients with overactive bladder symptoms [13, 14]. An inverse correlation between the intravesical concentration of ATP and bladder volume at FDV has previously been reported in two groups: patients with urodynamically proven DO [13] and patients with clinical OAB and low FDV [14]. However, the mechanism responsible for this was not elucidated.

Stretch is considered to be a major stimulus for ATP release in the bladder and it was hypothesized therefore that more ATP should be released as the bladder fills, with the highest ATP levels occurring at the end of the filling stage in accordance with murine studies which showed increased ATP release with increased intravesical pressure [15]. However, to our knowledge there are no reports indicating exactly when ATP is released during human bladder filling or how ATP release is influenced by the degree of bladder stretch. Therefore, we performed cystometry in women, collecting consecutive samples of bladder fluid at 200 mL and 400 mL and MCC for sequential ATP measurement. We recruited control patients and those with DO in order to characterise and compare the pattern of sequential ATP release in these two groups.

## 2. Materials and Methods

### 2.1. Cystometry

Each female patient was catheterised to empty the bladder completely for routine clinical cystometry [16], with the exception that dual lumen catheters were employed. A CSU was sent for culture and patients with proven bacterial cystitis (“UTI” 10^8^ cfu/L with pyuria >10/HPF) were excluded from subsequent analysis. Female patients gave informed consent in accordance with approval from the South Eastern Sydney human research ethics committee (SESIAHS HREC 06/11).

Saline at room temperature (approximately 25°C) was infused into the bladder at a filling rate of 75 mL/min (medium-fill) [16] as described previously [13]. As per routine, volumes were noted when the patient felt FDV and reached maximum cystometric capacity (MCC), (the volume at which the patient feels she can no longer delay micturition [16]), when filling was stopped. Tap water stimuli and erect provocation were routinely performed. At the end of cystometry, the patient voided the filling solution into a clean uroflow chamber, in private. The presence of any detrusor contractions during filling, or provocation such as the sound of running tap water or changing supine to erect position, was noted.

Patients were characterised as (a) idiopathic DO, with involuntary detrusor contractions during the filling phase which may be spontaneous or provoked, or (b) control, that is, pure urodynamic stress incontinence, involuntary leakage of urine during increased abdominal pressure in the absence of detrusor contractions, and no symptoms of urgency. Patients with the following were excluded: (a) bladder pain syndrome (FDV < 200 mL with bladder pain, and MCC < 400 mL), (b) voiding dysfunction (detrusor pressure (Pdet) at maximal flow greater than 60 cm H_2_O), (c) incomplete voiding (postvoid residual volume > 100 mL), or (d) evidence of spinal cord or central neurological disease.

### 2.2. Sampling and ATP Measurements

When the filling volume reached 200 mL, 400 mL, and MCC, the infusion was paused for sample collection. These volumes were chosen as fixed values that approximate the commonly observed filling volumes for first desire to void and maximal capacity in patients with DO (see discussion). At each volume, the first 10 mL in the collecting catheter (“deadspace”) was aspirated and discarded; then, a further 5 mL of intravesical filling fluid was collected. After voiding, fluid from the uroflow chamber was collected (known as “voided volume,” VV). Each woman thus yielded four samples for ATP assay. Immediately after collection, samples were transported on ice from the clinic to the laboratory in the adjacent building and then ATP assayed immediately.

ATP concentration (in nM) was measured in duplicate per sample, using a GloMax 20/20 luminometer and a bioluminescence assay (Sigma). A standard curve was constructed using freshly made standards (10^−5^ to 10^−10^ M and blank) from frozen concentrated ATP stock. The total amount of ATP (in nmoles) contained in each sample was then calculated by multiplying the filling volume (200 mL, 400 mL, MCC, or VV) by its corresponding ATP concentration.

### 2.3. Statistical Analysis

**Figure 1:**
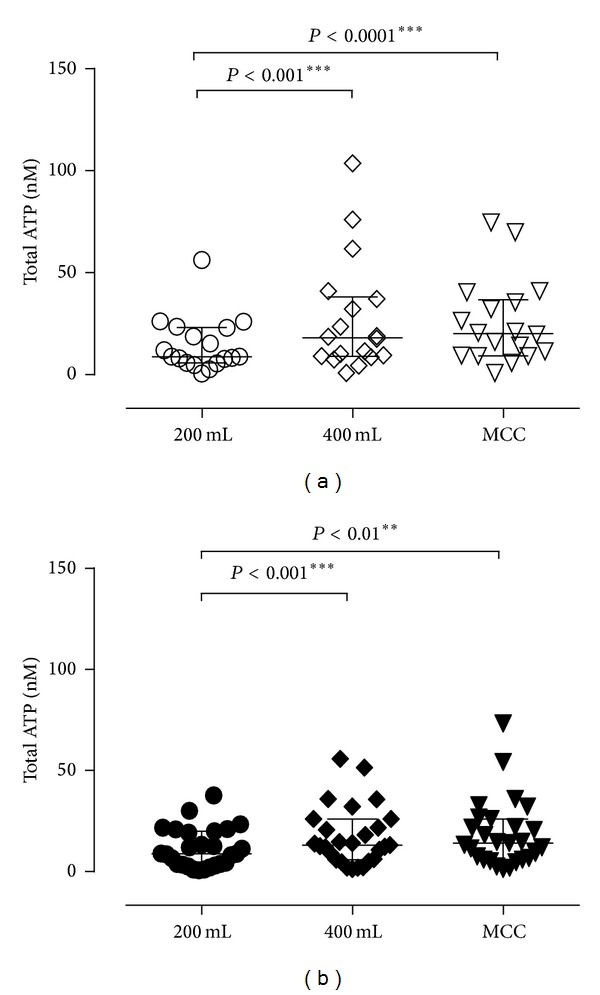
Comparison of total ATP (nmoles) in bladder fluid collected at volumes of 200 mL and 400 mL and at MCC, in control (*n* = 18) (a) and DO (*n* = 27) (b). ATP content significantly increased between filling volumes of 200 mL and 400 mL and between 200 mL and MCC. Data were analyzed by the Friedman test followed by Dunn's multiple comparison test. (** = *P* < 0.01; *** = *P* < 0.001).

**Figure 2:**
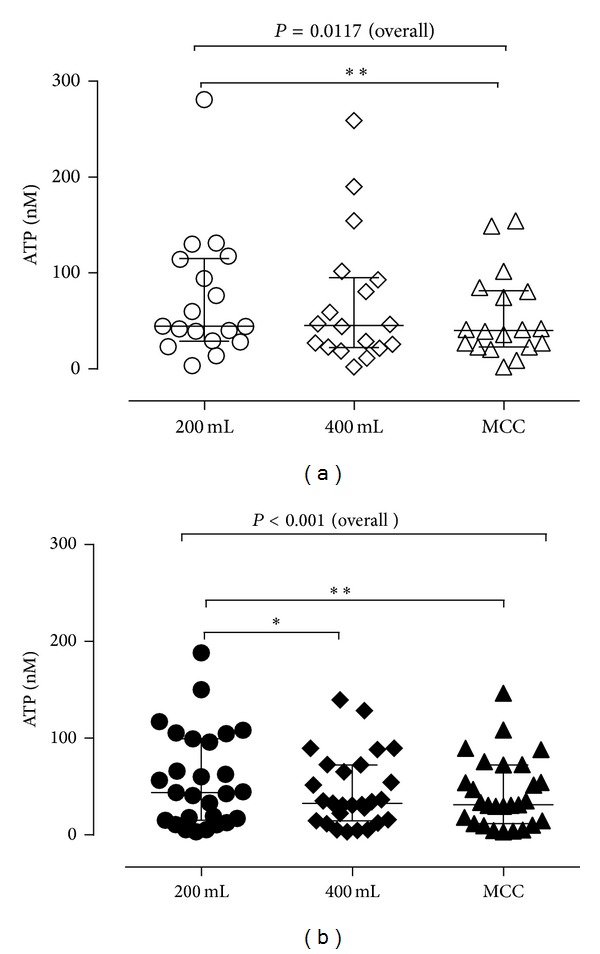
Comparison of ATP concentrations in bladder fluid collected at volumes of 200 mL and 400 mL and at MCC for control (*n* = 18) (a) and DO (*n* = 27) (b) patients. In this and subsequent figures, data points are from individual patients, showing median and interquartile range. Data were analyzed by the Friedman test followed by Dunn's multiple comparison test.

**Figure 3:**
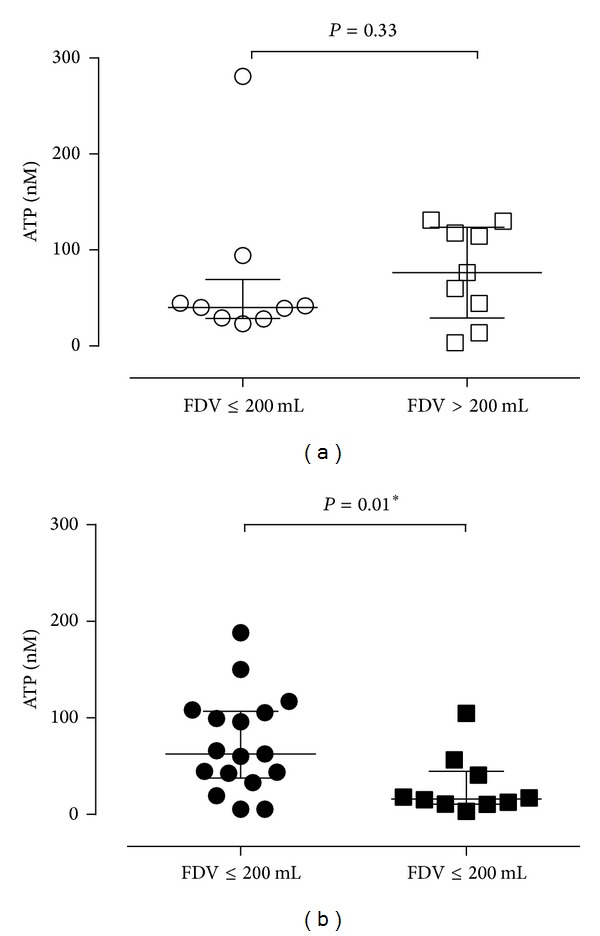
Comparison of ATP release at a bladder volume of 200 mL. For control (a) and DO patients (b), the data have been divided into two groups of low (≤200 mL) and higher FDV (>200 mL). The two control groups of low (*n* = 9) and high (*n* = 9) FDV showed no significant difference (*P* = 0.33), whereas there was a significantly higher concentration of ATP in the DO group with low FDV (*n* = 17), compared to the DO group with higher FDV (*n* = 10) (*P* = 0.01, Mann-Whitney test).

**Figure 4:**
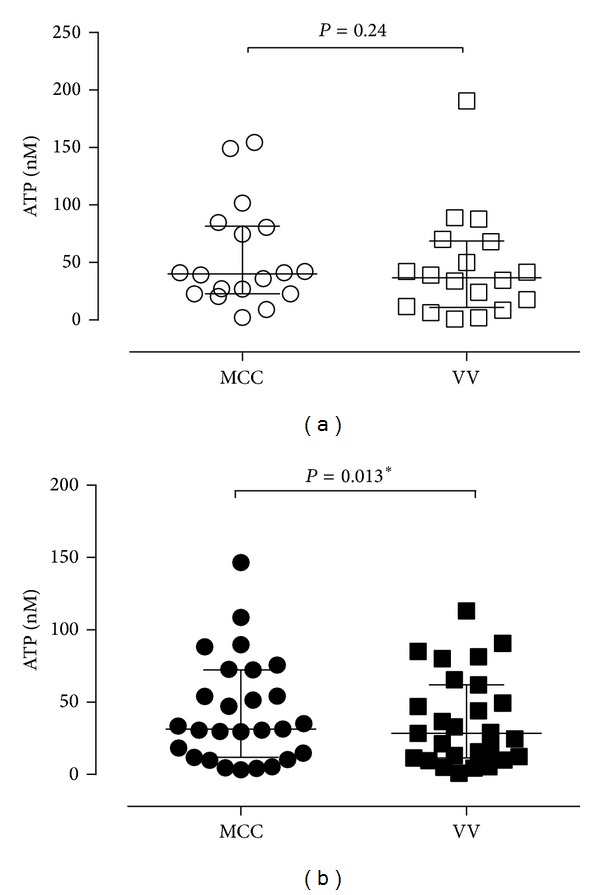
Comparison of ATP concentration before and after voiding in (a) control (*n* = 18) and (b) DO patients (*n* = 27). For control patients, there was no difference in ATP concentration of urodynamic fluid before MCC or after voiding (VV). For DO patients, there was a significant decrease in ATP concentration in VV compared with MCC (*P* = 0.01, Wilcoxon test).

ATP data were not normally distributed (*P* < 0.05, D'Agostino & Pearson omnibus normality test) and were expressed as median (interquartile range, IQR). Nonparametric statistical analyses were performed using GraphPad Prism 6.0 software (San Diego, USA). Comparison of ATP levels in the different samples was performed by Friedman ANOVA with Dunn's multiple comparisons test (Figures 1 and 4) or using a Mann-Whitney test when two unmatched groups were compared (Figure 2) or a Wilcoxon test when matched groups were compared (Figure 3). Methods, definitions, and units conform to the standards recommended by the International Continence Society except where specifically noted.

## 3. Results and Discussions

### 3.1. Stretching Is a Stimulate to Induce Further ATP Release in Both DO and Controls

Although ATP release is induced by urothelial stretch during bladder filling, it is unclear whether stretch-induced ATP is involved in triggering bladder contraction, either at physiological maturation or during pathological conditions such as DO, and the pattern of ATP release in human bladder and its association with bladder sensation have not been clearly understood. Traditional animal models cannot provide this information, because of the small bladder volume in rats and mice and the inability to assess the sensations of bladder filling in the rodent. Results from* in vitro* laboratory studies reporting ATP release from human bladder urothelium/lamina propria strips have shown a very marked increase in stretch-induced ATP release in DO, compared with control [12]. Using urodynamic fluid to investigate ATP release has become a valuable tool to answer these questions under true physiological condition relevant to patients.

To investigate how bladder stretch could induce ATP release, we also chose to collect samples at volumes of 200 mL, which approximates the FDV in most patients (median FDV in DO 167 mL and control 200 mL, Table 1), and a filling volume of 400 mL, which is close to the MCC (median MCC 430 mL) in most DO patients, in addition to sampling at MCC and in voided fluid. We compared ATP release in these consecutive intraluminal fluid samples from each patient. These intraluminal fluid samples have been generated during routine urodynamic testing under standard clinical conditions which occurs at a supraphysiological filling rate (75 mL/min) [16].

Cystometry with measurement of ATP at the four filling volumes was performed on 53 women (age 28–87 yrs). Eight subjects with UTI on the test day and 2 subjects with voiding dysfunction were excluded, yielding 27 patients with DO and 18 control patients (Table 1).

The total amount of ATP (in nmoles) at each bladder volume was examined. For both control (Figure 1(a)) and DO (Figure 1(b)), there was a significant increase in the total amount of ATP, in nmoles, between 200 mL and 400 mL, as well as between 200 mL and MCC; the total amount of ATP in the intraluminal fluid (in nmoles) was the lowest at the lowest bladder filling volume (total ATP median values (in nmoles) at 200 mL, 400 mL, and MCC are 8.8, 13.1, and 14, for DO, and 8.9, 18.1, and 20.2, for controls). Note that ATP was released continuously throughout the urodynamic test in both DO and control and that the total ATP content in the intraluminal fluid increased during filling, that is, from 200 mL to 400 mL, which agreed with the initial hypothesis that ATP release was induced by bladder stretch in both DO and controls.

We have also compared the difference in ATP release between DO and control groups at each filling/sampling point, but we did not see any significant difference between the two groups. One of the reasons for this could be the variations in ATP release in individuals, which made it too hard to see the difference between two groups. This is not a surprise as there was no significant difference in ATP release between DO and control patients with a much larger sample size in a previous study [13].

### 3.2. Substantial ATP Released at the Early Bladder Filling Stage

We also compared the ATP concentrations according to the filling volumes and determined that in both control (Figure 2(a)) and DO (Figure 2(b)) patients, there was a significant difference in ATP concentration at the three different bladder volumes (Friedman test). Overall, in both control and DO patients the ATP concentration was higher at the early filling stage (200 mL, median ATP 44.5 nM control; 43.8 nM DO) compared with later stages (MCC, median ATP 40.2 control; 31.3.7 nM DO).

This is a surprising and important finding. This indicates that there was substantial ATP release at early filling volumes in both DO and controls, and early ATP release may play an important role in bladder function and dysfunction, that is, early sensation or urgency for DO.

### 3.3. Early ATP Concentration Is Related to the First Desire Volume (FDV) in DO Patients

In the DO patients, the median FDV was 167 mL, with values ranging between 85 and 400 mL. In order to investigate whether FDV was associated with ATP release, patients were divided into two groups based on low FDV (≤200 mL) and higher FDV (>200 mL). In control patients, no difference in ATP concentration in intravesical fluid at 200 mL was seen between the two groups (FDV less than 200 mL (ATP median = 40.2 nM) and FDV more than 200 mL (ATP median = 76.5 nM)) (Figure 3(a)). However, in DO patients, the concentration of ATP in intravesical fluid at 200 mL was significantly higher in patients with FDV ≤ 200 mL than those with FDV > 200 mL (Figure 3(b)). The same analysis was applied to the data sets from the 400 mL, MCC, and VV samples, but no association was shown between FDV and ATP release at these volumes (data not shown).

The fact that DO patients with a lower FDV (≤200 mL) had higher ATP concentrations (ATP median 62.7 nM) at an earlier bladder volume, compared with DO patients who had a higher FDV (>200 mL) (ATP median 16.1 nM), suggests that the early ATP release may play an important role in DO patients, which may cause urgency. The previous clinical studies have also found that ATP in voided urodynamic fluid was inversely correlated with the FDV in DO [13] and OAB [14] patients, but not controls. This current study supports the supposition that it is the early ATP release which is important to the sensation of urgency in DO patients.

Although ATP can be degraded by membrane-bound ectoATPases, studies have shown that there is little ectoATPase activity associated with the apical surface of the urothelium [17]; thus the intravesical ATP measured may reflect the ATP released from the urothelial cells. Moreover, the source of the ATP is assumed to be from the urothelial cells, but this was not able to be determined from our* in vivo* study. The ATP in bladder intraluminal fluid might be released directly from urothelial cells into the lumen and/or could diffuse into the lumen from various sources within the bladder wall, such as from the suburothelial myofibroblasts. Diffusion into the lumen from suburothelial cells would be limited by the intact bladder urothelium and also by the ectoATPase activity associated with intermediate and basal urothelial cells and also with the lamina propria [17]. However, a reduced ectoATPase activity in laboratory samples of OAB has been reported [18]; thus, more ATP may diffuse into deep sites of bladder wall, enhancing stretch-induced signalling pathways inducing urgency or involuntary contractions. These findings may explain why the early ATP release plays an important role in early sensation (urgency) in DO but not in controls.

### 3.4. Voiding Did Not Further Induce ATP Release

Previous studies have reported that ATP plays a role in the bladder as a purinergic neurotransmitter in functional motor [6] as well as sensory bladder disorders [12]. Thus ATP might also appear in the bladder as a result of parasympathetic nerve stimulation to the detrusor during voiding [19].

In order to investigate whether a micturition contraction might increase ATP release in the bladder, we compared the ATP concentration before voiding (MCC) and after voiding (VV). The results showed no significant difference in ATP concentration between MCC (ATP median = 40.2 nM) and voided volumes (ATP median = 36.8 nM) in control patients (Figure 4(a)); that is, ATP content remained unchanged after voiding. In DO patients (Figure 4(b)), there was no evidence of any increase in ATP release and in fact the ATP concentration was slightly lower in voided fluid (VV) (ATP median = 28.4 nM) than in bladder intraluminal fluid at MCC (ATP median = 31.3 nM) (*P* = 0.01, Wilcoxon test). This suggests that the voiding contraction does not contribute further to intravesical ATP release in bladder lumen.

This is not surprising as ATP released from motor nerves will be deep in the detrusor muscle layer and unlikely to reach the bladder lumen due to abundant ecto-ATPase in lamina propria and smooth muscle [17]. Instead of increased ATP after voiding, we have noticed a decreased intravesical ATP after voiding in DO patients in our study. It is possible that, during micturition, the intravesical ATP is exposed to ectoATPase located on exfoliated urothelial cells [20] and, therefore, ATP may undergo enhanced degradation during or after voiding.

## 4. Conclusions

For the first time ATP release during bladder filling has been investigated in sequential intraluminal fluid samples in female patients with or without DO. This study has shown that total amount of ATP in intraluminal fluid (in nmoles) continued to increase from lower bladder volume (200 mL) to higher bladder volume (400 mL and MCC) in both DO and controls. The study demonstrates that stretch (bladder filling) can induce further ATP release in human bladder. Interestingly, we have noticed that there was a substantial ATP release in intraluminal fluid in the early filling stage, that is, bladder filling volume of 200 mL. The results also show that ATP release at 200 mL filling volume is related to FDV only in DO patients but this relationship was not observed in the control group. These data indicate that ATP release at early bladder filling may play an important role in increased bladder sensations, for example, urgency, in DO patients.

## Figures and Tables

**Table 1:** Patient urodynamic characteristics.

	Control (median (IQR))	DO (median (IQR))
*n*	18	27
Age (years)	60.5 (47–70)	59 (54–66)
FDV (mL)	200 (170–265)	167 (100–277)
MCC (mL)	492 (450–500)	430 (400–500)
*Max Pdet during filling (cm H_2_O)	6.5 (5–9.25)	33 (17–46)
Detrusor contractions during cystometry	0	27
VV (mL)	485 (415–532.5)	417 (380–500)

*Maximal detrusor pressure during filling or provocation.

## Abbreviations

ATP: Adenosine triphosphate
CSU: Catheter specimen of urine
DO: Detrusor overactivity
FDV: First desire to void
MCC: Maximal cystometric capacity
Pdet: Maximal detrusor pressure
VV: Voided volume.
